# Ecotone analysis: assessing the impact of vehicle transit on saltmarsh crab population and ecosystem

**DOI:** 10.1186/2193-1801-3-655

**Published:** 2014-11-05

**Authors:** Claudia Trave, Marcus Sheaves

**Affiliations:** School of Marine and Tropical Biology, James Cook University, Townsville, Qld 4811 Australia; TropWATER (Centre for Tropical Water & Aquatic Ecosystem Research), James Cook University, Townsville, Qld 4811 Australia

## Abstract

The frequent transit of vehicles (recreational or not) through saltpans and saltmarsh fields has been recorded as one of the major causes of physical and ecological damage for these environments. While several studies have been carried out to assess the consequence of this anthropogenic activity on the different local plant species, little is known on its long-term impact on the faunal community. Invertebrates, such as crabs, provide several essential ecological services, and their presence and abundance are tightly connected to that of the saltmarsh plants. Decrease of vegetative cover due to vehicle transit is likely to cause alterations in the morphology and the composition of the saltmarsh ecosystem. In this study we evaluate presence and distribution of the main crustacean species in several impacted sites in Townsville area (Queensland, Australia), to determine possible correlation between vehicle tracks alterations and crab distribution, as well as investigate any possible habitat shift in the mid- and long-term. Results indicate that reduction of plant cover affects species composition and distribution, with different effects based on the unique characteristics of each crab species analysed, resulting in an overall alteration of the assemblage structure.

## Introduction

In tropical and subtropical areas, estuaries comprise a complex mosaic of mangrove forests, seagrass beds, salt pans, saltmarshes, mud/sand flats and intertidal shores. Although structurally quite different, these habitats are tightly interconnected and interdependent; consequently, damage or alteration to one may flow on to cause substantial or even irreversible changes to others. (Kennish 2002; Lee *et al*. 2006; Valiela and Cole 2002).

Studies of environmental threats have mainly focussed on mangrove and subtidal habitats (Alongi 2002; Duarte 2002; Valiela *et al*. 2001), with much less attention paid to tidal marsh ecosystems; often overlooked for their apparent low species diversity and lack of intensive biological activity. However, saltmarshes play a key role in sustaining local biodiversity and productivity (Abrantes and Sheaves 2009), so their health is of fundamental importance for the welfare of neighbouring habitats and the human communities that rely on them (Bromberg-Gedan *et al.*^2009^; Valiela and Cole 2002). Saltmarshes provide temporary or permanent habitat for a variety of species. When flooded they offer shelter and foraging grounds for juvenile fish and other aquatic organisms but during low tide they are populated by wetland and terrestrial species – mostly invertebrates – that rely on saltmarsh plants for shade, foraging, burrowing and predator avoidance (Shenker and Dean 1979; Burger and Lesser 1979; Nomann and Pennings 1998).

Like other wetland ecosystems, tidal marshes are increasingly impacted by anthropogenic activities; it has been estimated that approximately 50% of the original saltmarshes have been lost or damaged globally (Bromberg-Gedan and Silliman 2009; Bromberg-Gedan *et al.*^2009^). Among the causes of this habitat degradation are reclamation of land for crops and constructions (Bromberg-Gedan and Silliman 2009; Finlayson and Rea 1999; Kennish 2001; Laegdsgaard 2006), alteration of local hydrology (Craig *et al*. 1979; Davis and Froend 1999; Deegan *et al.*^1984^), pollution (Davis and Froend 1999), waste disposal (Bromberg-Gedan and Silliman 2009; Deegan *et al.*^1984^), stock grazing (Andresen *et al.*^1990^; Finlayson and Rea 1999), and trampling (Finlayson and Rea 1999).

While bunding and reclamation have caused major reductions in their areal extent Sheaves et al. (2014), tropical saltmarshes are also threatened by local-scale activities, such as vehicle passage; one of the major causes of physical and ecological damage in saltmarsh areas (Blionis and Woodin 1999; Kelleway 2006). The constant transit of cars, four-wheel drives and bikes causes damage to soil structure and decreases vegetation coverage, leading to the formation of trails or empty stretches of bare earth. Any decrease of vegetative cover or alterations in plant species presence/dominance is likely to change the morphology, composition and functioning of the whole saltmarsh ecosystem, with the potential of fragmentation of animal as well as plant communities. Although the effects of such disturbances have been investigated for saltmarsh plants, little is known of impacts on faunal communities. Kelleway (2006) investigated gastropod shell occupancy and crab burrow density in temperate Australia but did not investigate faunal responses at the species level. Beyond this there have been few studies of impacts on saltmarsh fauna and none in the tropics. However, strong, and often obligate plant/animal relationships are common across many tropical wetland types (Nagelkerken *et al*. 2008), meaning the presence and abundance of resident animals, such as crabs, is often tightly connected to that of the local plants. In fact, in temperate regions, a range of specialist saltmarsh organisms depend on the presence of saltmarsh plants for their survival (Nomann and Pennings 1998), so a similar situation would be expected in tropical marshes.

We evaluated the effects of chronic vehicle passage on tropical saltmarsh ecosystems, with particular focus on the consequences of alteration of habitat for semi-terrestrial crabs. To do this we evaluated changes in vegetation cover and sediment characteristics between impacted and non-impacted areas, and determined differences in crab species composition, distribution and habitat utilization (by assessment of burrow occurrence).

## Materials and methods

### Study areas

**Figure 1:**
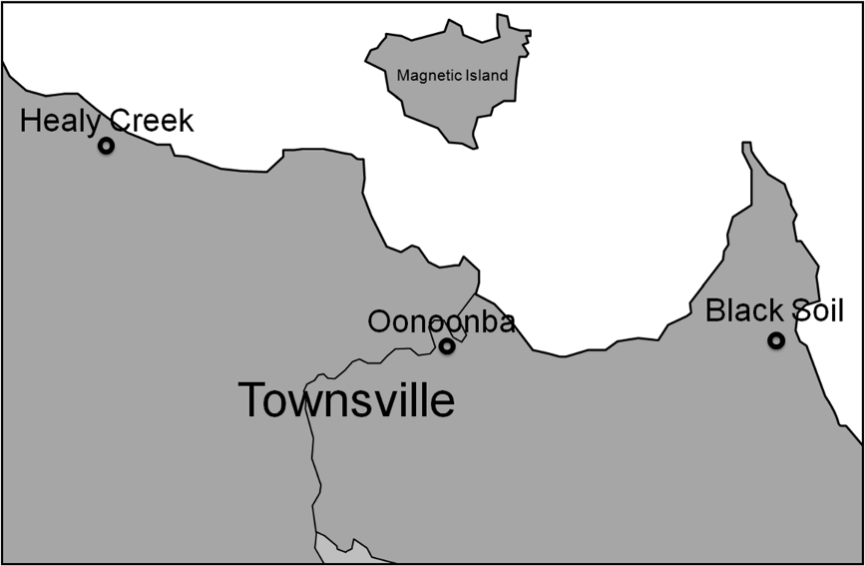
**Map of study areas.**

The study was conducted at three estuarine areas in Townsville, tropical north Queensland, Australia – Black Soil Creek (BS), Oonoonba (ON) and Healy Creek (HC) (Figure 1). Each area was characterised by extensive saltmarsh and saltpan fringed by mixed mangrove forests on their seaward margins. No significant differences in elevation between the various habitats (and within the saltmarsh field itself) that might influence flora and fauna presence/distribution were recorded. The study areas chosen were commonly used for recreational activities (i.e. BMX, trailbikes and quadbikes). In each location constant passage of vehicles through the saltmarshes had caused the formation of recognisable trails devoid of vegetation.

From the analysis of historical aerial photographic record (Google Earth Maps) of the ecology and habitat composition of the study areas it was possible to determine prior to the field sampling the presence of saltmarsh vegetation (be it full cover or patchy) in the areas where the tracks are currently located, thus highlighting any change as human induced. Sparsely vegetated areas were discarded for this study as the apparent lack of vegetation could not be attributed with certainty to the transit of vehicles over time.

Two study sites were investigated at each area to account for local variability: Black Soil 1 (BS1), Black Soil 2 (BS2), Oonoonba 1 (ON1), Oonoonba 2 (ON2), Healy Creek 1 (HC1), and Healy Creek 2 (HC2).

### Sample collection, processing and analysis

**Figure 2:**
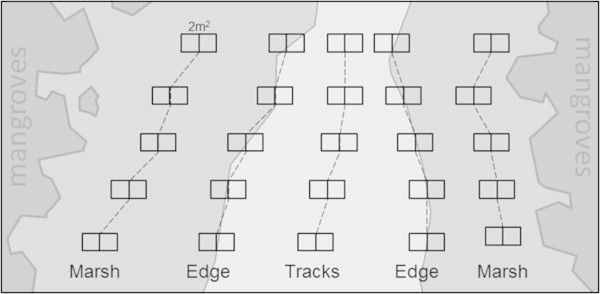
**Example of study sectors and quadrats disposition.** The colour gradient used in this image represents the different habitats: from darker to lighter respectively mangrove forest, saltmarsh field and bare areas of vehicle tracks.

Data were collected from five sectors in each study site: two within the saltmarsh field at least 2 meters from the nearest track (‘Marsh’), two along the edge of the marsh fringing the tracks (‘Edge’) and one within the track paths (‘Tracks’) (Figure 2). In each sector five 2 m × 1 m quadrats were randomly placed lengthwise. Quadrats in ‘Edge’ sectors were placed so that 1 m^2^ would cover the grass (‘inner’) and 1 m^2^ would extend onto the track edge (‘outer’). Sampling was undertaken at mid-low tide when the areas were not inundated and at least 24 h after any rainfall event. The presences of flora, fauna, and burrow density were recorded though visual observation and, when required, capture.

Saltmarsh plants were identified at species level and their overall presence/absence recorded, as an indication of species dominance and relative abundance: 5quadrats × 4 sectors × 2sites for a total of forty assessments per study area (Oonoonba, Black Soil and Healy Creek).

Manual capture of crabs and visual census were performed to determine species composition and their distribution along the three sectors. All burrows located in the sampling quadrats were inspected, as several individuals were found to remain close to the opening or hiding among the plant stems. In addition, visual observations were carried out from a distance of 3-4 m after a waiting time of 10-15 min – the minimum time recorded on average for the animals to come out in the open after a disturbance. The combination of these sampling methods allowed to accurately determine the presence and distribution of the different crab species, and compensate for the disadvantages/obstacles typical of each technique (Mazumder 2009; Mazumder and Saintilan 2010).

To assess the degree of habitat utilization on behalf of the local crab population as a whole, crab burrows were counted at each quadrat by moving (when present) saltmarsh foliage and stems to allow an unobstructed view of the ground and perform an accurate and thorough assessment.

Soil compaction was estimated from twenty penetrometer measurements (four per sector) at each site. Two replicates were performed per each study area.

### Statistical/Data analyses

The overall presence/absence of the saltmarsh species recorded for the ‘Marsh’ and ‘Edge’ sectors at each of the three study areas was plotted in a histogram, to display the composition of the saltmarsh fields and relative distribution of the plant species identified.

Burrow density values were transformed (fourth root) to produce approximately homogeneous variances and a Multi-factorial Analysis of Variance (ANOVA) performed to test for differences among the three sectors (Marsh, Edge and Tracks) in the study sites, with the three study sites and three sectors as predictor variables.

Classification and Regression Trees (CART) were constructed for *Sesarma longicristatum* and *Uca signata* to determine whether the different conditions between the three sectors influence the distribution of the two main crab species. Dependant variable: presence/absence of crabs. Predictor variables were: Site = ON, BS, HC; Sector = Marsh-Edge-Track gradient; and Penetrability = cm of penetration in/through the top soil.

## Results

**Figure 3:**
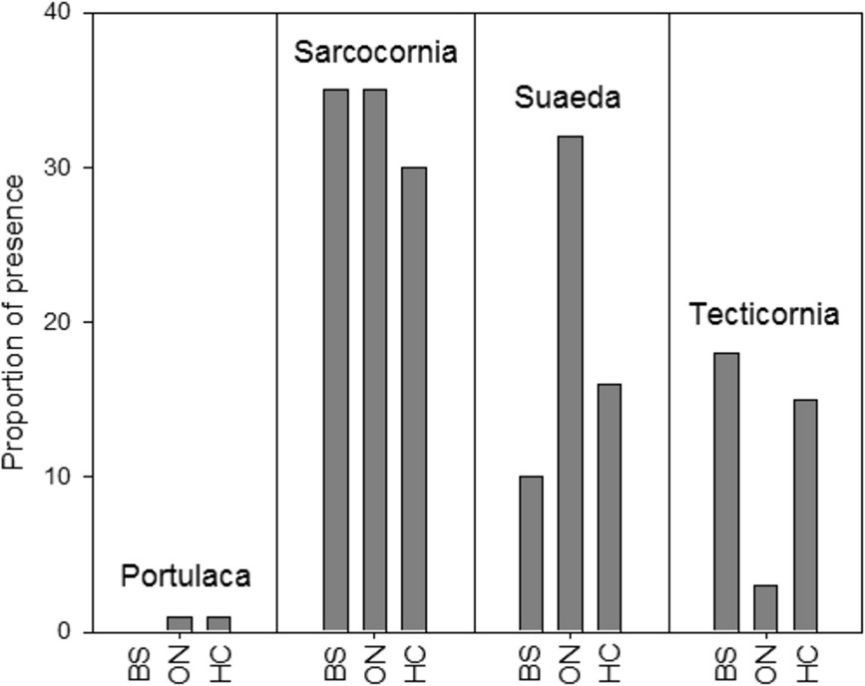
**Overall proportional presence of the succulent plant species in the three study areas: presence/absence out of 40quadrats per study area.**

Eight plant and five crab species were identified in the saltmarsh ecosystems at Black Soil, Oonoonba and Healy Creeks (Table 1). Succulent plants were the main component of the saltmarsh vegetation in all three areas, with *Sarcocornia quinqueflora* and *Suaeda australis* as the dominant species, and *Tecticornia indica* together with *Portulaca oleracea* present in lesser densities (Figure 3).

**Table 1:** **Species identified within the saltmarsh in the three study sites**

Species	Black Soil	Oonoonba	Healy Creek
Succulent plants			
*Portulaca oleracea*		x	x
*Sarcocornia quinqueflora*	x	x	x
*Suaeda australis*	x	x	x
*Tecticornia indica*	x	x	x
Grass			
*Sporobolus virginicus*		x	x
Mangroves			
*Aegialitis annulata*		x	x
*Avicennia marina*	x	x	x
*Ceriops spp.*			x
Crabs (Grapsoidea)			
*Metopograpsus latifrons*		x	
*Sesarma longicristatum*	x	x	x
Crabs (Ocypodoidea)			
*Australoplax tridentata*		x	
*Cleistostoma wardi*	x	x	
*Uca signata*	x	x	x

Three mangroves and one grass species were found occasionally interspersed among the succulent plants. Although the surrounding mangrove forests appeared uniform and thick, within the saltmarsh fields mangroves were found only as single shrubs or saplings, often several meters distant from one another. The mangroves *Aegialitis annulata* and *Avicennia marina* occurred at most study areas, while *Ceriops spp.* was recorded only in Healy Creek, and at a very low density (Table 1). The grass *Sporobolus virginicus* was recorded in Oonoonba and Healy Creek but not at Black Soil Creek (Table 1). Vehicle tracks were clearly visible at all sites marked by bare ground criss-crossed by deep (3-25 cm) ruts. There were no seedlings or signs of regrowth in the ruts created by the vehicles.

Five crab species where identified in the three study areas (Table 1).

*Sesarma semperi longicristatum*, *Australoplax tridentata, Cleistostoma wardi* and *Metopograpsus latifrons* were found only within the saltmarsh field, while fiddler crab *Uca signata* was observed in areas devoid of vegetation as well as in the saltmarsh, particularly where tyre ruts retained water from tidal inundation. Of the five species found in this study, only *U. signata* and *S. longicristatum* were found in adequate abundance to allow further analyses.

**Figure 4:**
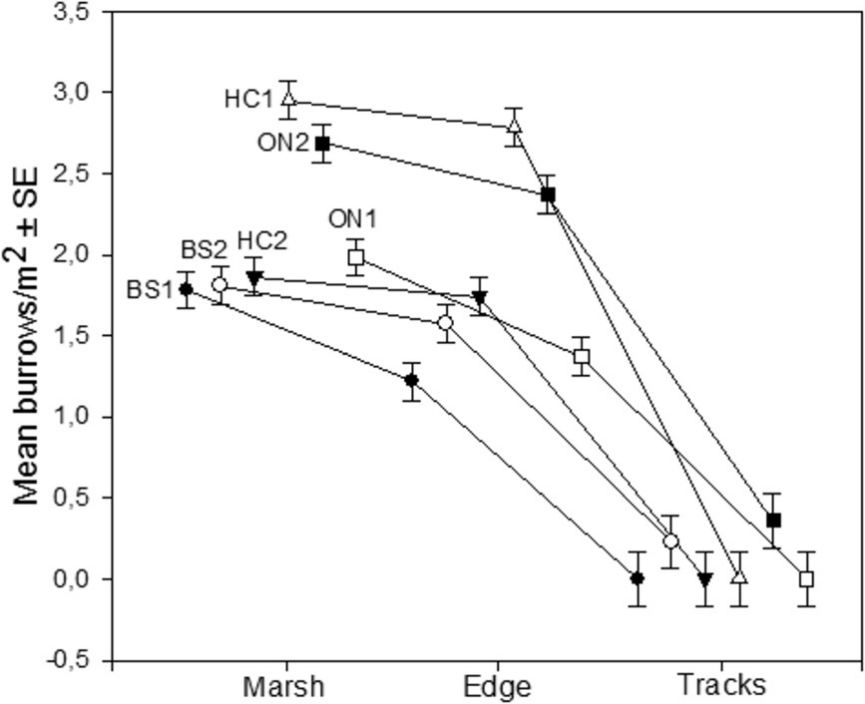
**Mean crab burrows density measured in the three sectors (Marsh, Edge and Tracks) for all study sites.**

At all sites crab burrows decreased from the marsh field toward the car tracks (F = 4.9676, df = 10/282 ; p = 0.0000; CI = 0.95) (Figure 4). While values obtained for the saltmarsh fields and edges varied among the different sites, the ‘Tracks’ sector showed little (BS2 and ON2) or no sign of crab burrows (BS1, ON1, HC1 and HC2).

**Figure 5:**
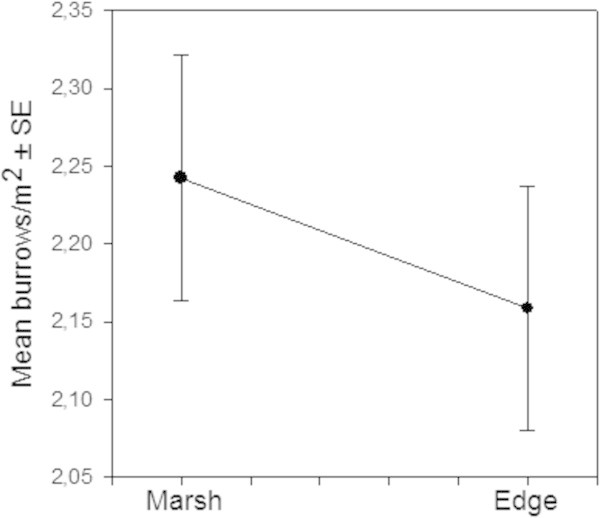
**Edge effects analysis: Values of burrow density per m** ^**2**^ **were compared between the ‘Inner’Edge and Marsh sector for all study sites to determine significant differences.**

**Figure 6:**
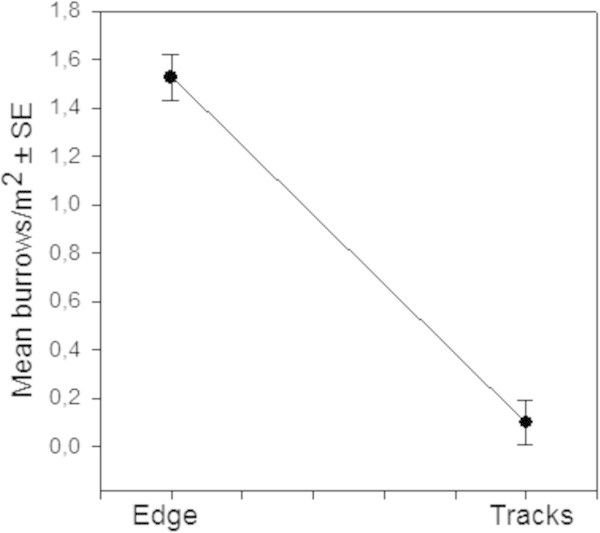
**Edge effects analysis: Values of burrow density per m2 were compared between the ‘Inner’Edge and Marsh sector for all study sites to determine significant differences.**

To determine possible edge effects, values recorded for ‘inner’ quadrats from the Edges sectors were compared to those measured in Marsh, while those from the ‘outer’ quadrats were compared to the values recorded in the Track sector (Figures 5 and 6).

No significant difference was observed between the average number of burrow estimated for ‘inner’ Edges and Marshes (F = 0.568, df = 1/118 ; p = 0.452; CI = 0.95), for either Left or Right sides. In contrast, there were clear differences in burrow density between the saltmarsh Edges and Tracks (F = 117.25, df = 1/118 ; p = 0.0000; CI = 0.95). While mean burrow density was close to zero in the sector where vehicle traffic was recorded, average values in the adjacent edges of the saltmarsh field ranged between 1.46–1.60 burrows/m^2^ (Figures 5 and 6).

**Figure 7:**
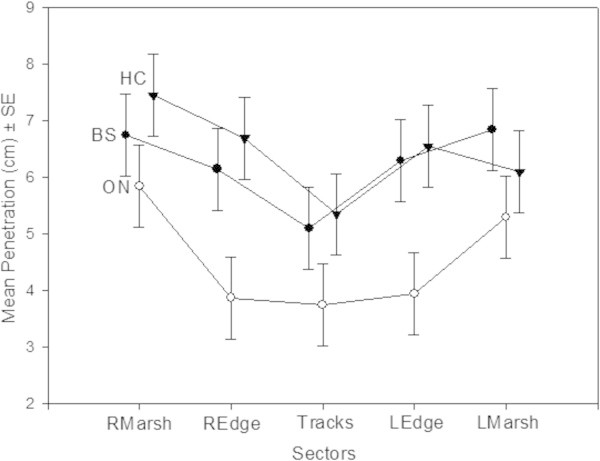
**Mean soil values of penetration depth measured in the different sectors of the three study areas: Black Soil, Oonoonba and Healy Creek.**

While penetrometer depth varied among study sites, there was always an increase of compaction (lower penetration) along the Marsh-Track gradient (F = 6.982, df = 2/111 ; p = 0.001; CI = 0.95) (Figure 7).

**Figure 8:**
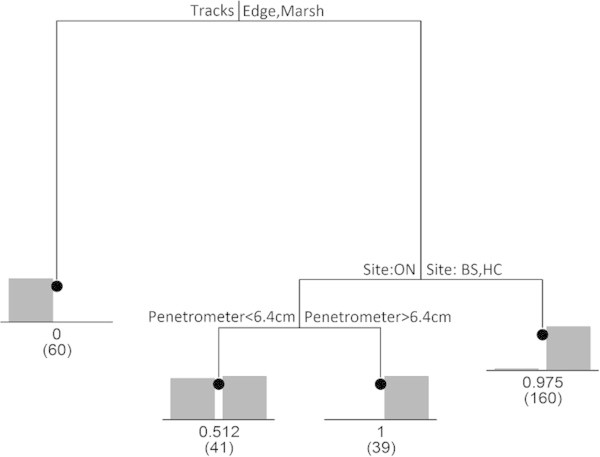
**Classification and regression tree (CART) of** ***S. longicristatum*** **distribution in the study areas.** The splits in the tree occur at the points of maximum dissimilarity of presence/absence.

CART analysis showed different factors influenced the distributions of *S. longicristatum* and *U. signata* (Figure 8). No *S. longicristatum* individuals were found in the Tracks sector but occurrences were similar for Edge and Marsh habitats. For the Edge/Marsh group, presence of *S. longicristatum* resulted higher at Black Soil and Healy Creek than the one recorded at Oonoonba. Additionally, there was no detectable influence of soil compaction on distribution at Black Soil and Healy Creek and almost all Edge/Marsh replicates contained *S. longicristatum*. However, at Oonoonba it was observed that where the sediment was more compact (penetrometer depth <6.4 cm) only half the quadrats were found to contain crabs (Figure 8).

**Figure 9:**
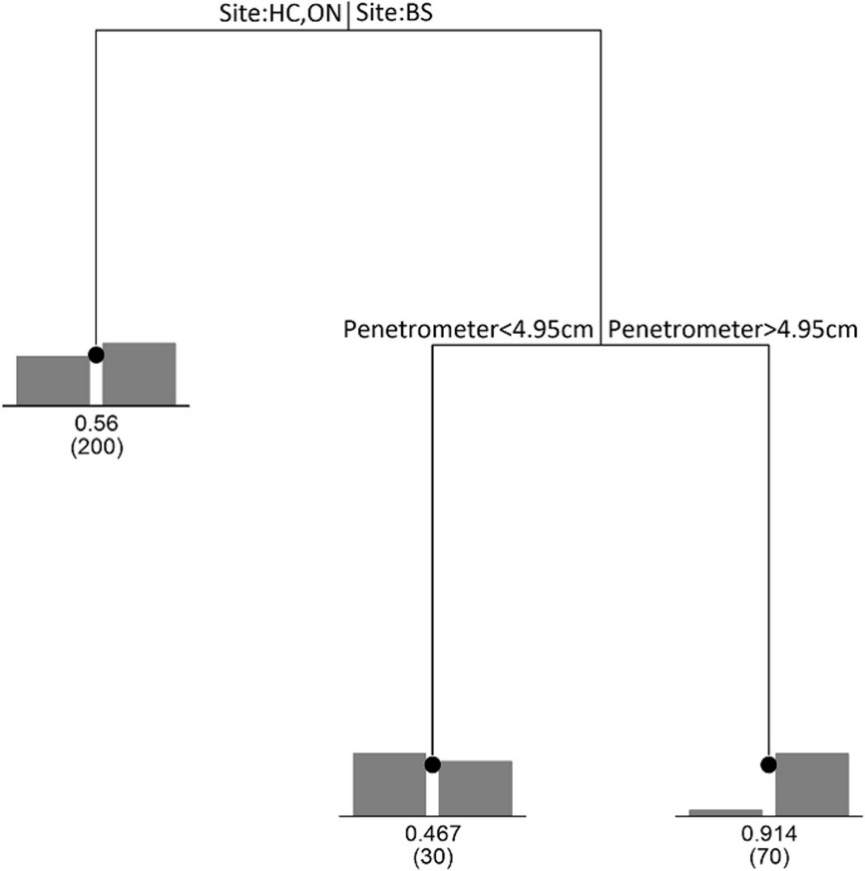
**Classification and regression tree (CART) of** ***U. signata*** **distribution in the study areas.** The splits in the tree occur at the points of maximum dissimilarity of presence/absence.

In contrast, the occurrence of *U. signata* did not differ among sectors (Figure 9), with most variation in occurrence taking place among the three study areas. Again compaction produced a subsidiary split with *U. signata* occurring in most quadrats where sediment compaction was low (penetrometer depth >4.95 cm) but only in about half the quadrats where compaction was high.

## Discussion

Vehicle transit severely impacts the landscape of saltmarsh ecosystems, causing immediate (and often irreversible) damage to individual plants and gradual vegetative coverage loss, especially in those areas where vehicle passage is a chronic event (Blionis and Woodin 1999; Kelleway 2006). This expected loss of marsh vegetation was obvious at all three study sites, and was matched by strong differences in crab assemblages and burrow densities between the vegetated marsh surface and areas devoid of vegetation because of vehicle passage.

The presence of vehicle track-ways did not only affect the total number of organisms but had a strong influence on assemblage structure. Of the 5 species found at the study sites, only one, *Uca signata,* inhabited the unvegetated track-ways; while *S. longicristatum*, *A. tridentata. C. wardi* and *M. latifrons* were found only in the saltmarsh fields – mostly *Sarcocornia*, *Tecticornia* and *Suaeda*. Most intertidal crab species rely on the presence of vegetation cover for their survival (Nomann and Pennings 1998), so the limited food resources, higher exposure to predators and heat, would render the bare track-ways less hospitable to crabs. Indeed, burrow counts decreased significantly from the marsh field toward the track edges, and completely disappear in the track-way areas (Figure 5), probably a result of the increase in sediment density associated with vehicle passage (Kelleway 2006). The soil compacted by the passage of vehicles makes it more difficult for crabs to penetrate when digging their burrows (Bertness and Miller 1984), particularly in those areas with low frequency/duration of tidal inundation.

Differential effects on different crab species probably reflect their distinct habitat and environmental requirements. Depending on species, the conversion to unvegetated sediment either reduced the available habitat and altered the conditions required for their survival (*S. longicristatum*, *A. tridentata. C. wardi* and *M. latifrons*) or created new stretches of land to colonise (*U. signata*), with little competition from other species. In the long term, this is likely to lead to a radical environmental shift from a vegetated habitat with high species diversity to an unvegetated saltpan/mudflat populated only by fiddler crab *U. signata*.

The progressive decrease in overall crab abundance that appears likely to result from loss of saltmarsh vegetation would have repercussions on the remaining plants as well. Crab burrowing contributes to plant health by increasing soil aeration, drainage, and by facilitating the decomposition of plant debris (Bertness 1985; Montague 1982). A significant reduction of these macrobenthic invertebrates is likely to result in a bottom up effect leading to a decline in grass production.

Changes in species composition and/or abundance would also affect the structure of the local food web, with ripple effects propagating to neighbouring ecosystems. With their function as detritivores and mediators between primary producers and higher trophic levels, intertidal crabs play a fundamental role in estuarine food webs (Bouillon *et al*. 2002; Vermeiren and Sheaves 2014). Any alteration in biodiversity for both plants and animals would lead to progressive changes in the saltmarsh ecosystem structure and functioning (Chapin *et al.*^1997^). Such changes might also have repercussions on the resilience of the entire system, increasing its vulnerability to external stressors, reducing its stability, and its ability to recover (Peterson *et al.*^1998^) or to keep up with possible competing ecosystems - e.g. progressive mangrove transgression into saltmarsh areas (Saintilan and Williams 1999).

In the long term this situation could have dire consequences for the saltmarsh ecosystem and, by proxy, have repercussions to the neighbouring tidal habitats. It is therefore of utmost importance to develop measures to prevent a radical loss of saltmarshes and reduce human-induced stress and damage – such as that caused by vehicle transit – through the creation and implementation of *ad hoc* laws and regulations.

### Ethics statement

All measurements have been carried out on public land and complying with local laws.

No protected species were sampled in this study.

## References

[CR1] Abrantes K, Sheaves M (2009). Sources of nutrition supporting juvenile penaeid prawns in an Australian Dry Tropics estuary. Mar Freshwater Res.

[CR2] Alongi DM (2002). Present state and future of the world’s mangrove forests. Environ conserv.

[CR3] Andresen H, Bakker JP, Brongers M, Heydemann B, Irmler U (1990). Long-term changes of salt marsh communities by cattle grazing. Vegetatio.

[CR4] Bertness MD (1985). Fiddler crab regulation of *Spartina alterniflora* production on a New England salt marsh. Ecology.

[CR5] Bertness MD, Miller T (1984). The distribution and dynamics of *Uca pugnax* (Smith) burrows in a new England salt marsh. J Exp Mar Biol Ecol.

[CR6] Blionis GJ, Woodin SJ (1999). Vehicle track damage to salt marsh soil and vegetation at Culbin Sands, NE Scotland. Transactions and Proceedings of the Botanical Society of Edinburgh and Botanical Society of Edinburgh Transactions.

[CR7] Bouillon S, Koedam N, Raman A, Dehairs F (2002). Primary producers sustaining macro-invertebrate communities in intertidal mangrove forests. Oecologia.

[CR8] Bromberg-Gedan K, Silliman BR, Silliman BR, Grosholz T, Bertness MD (2009). Patterns of salt marsh loss within coastal regions of North America: pre-settlement to present. Human impacts on salt marshes: a global perspective.

[CR9] Bromberg-Gedan K, Silliman BR, Bertness MD (2009). Centuries of human-driven change in salt marsh ecosystems. Ann Rev Mar Sci.

[CR10] Burger J, Lesser F (1979). Breeding behavior and success in salt marsh Common Tern colonies. Bird-Banding.

[CR11] Chapin FS, Walker BH, Hobbs RJ, Hooper DU, Lawton JH, Sala OE, Tilman D (1997). Biotic control over the functioning of ecosystems. Science.

[CR12] Craig NJ, Turner RE, Day JW (1979). Land loss in coastal Louisiana (USA). Environ Manage.

[CR13] Davis JA, Froend R (1999). Loss and degradation of wetlands in southwestern Australia: underlying causes, consequences and solutions. Wetl Ecol Manag.

[CR14] Deegan LA, Kennedy HM, Neill C (1984). Natural factors and human modifications contributing to marsh loss in Louisiana’s Mississippi River deltaic plain. Environ Manage.

[CR15] Duarte CM (2002). The future of seagrass meadows. Environ conserv.

[CR16] Finlayson CM, Rea N (1999). Reasons for the loss and degradation of Australian wetlands. Wetl Ecol Manag.

[CR17] Kelleway J (2006). Ecological impacts of recreational vehicle use on saltmarshes of the Georges River, Sydney. Wetlands (Australia).

[CR18] Kennish MJ (2001). Coastal salt marsh systems in the US: a review of anthropogenic impacts. J Coastal Res.

[CR19] Kennish MJ (2002). Environmental threats and environmental future of estuaries. Environ conserv.

[CR20] Laegdsgaard P (2006). Ecology, disturbance and restoration of coastal saltmarsh in Australia: a review. Wetl Ecol Manag.

[CR21] Lee SY, Dunn RJK, Young RA, Connolly RM, Dale PER, Dehayr R, Welsh DT (2006). Impact of urbanization on coastal wetland structure and function. Austral Ecol.

[CR22] Mazumder D, Saintilan N (2009). Ecology of burrowing crabs in temperate saltmarsh of south-east Australia. Australian Saltmarsh Ecology.

[CR23] Mazumder D, Saintilan N (2010). A comparison of sampling techniques in the assessment of burrowing crab abundance in saltmarsh and mangrove environments. Wetlands (Australia).

[CR24] Montague CL, Kennedy VS (1982). The influence of fiddler crab burrows and burrowing on metabolic processes in salt marsh sediments. Estuarine Comparisons.

[CR25] Nagelkerken I, Blaber SJM, Bouillon S, Green P, Haywood M, Kirton LG, Somerfield PJ (2008). The habitat function of mangroves for terrestrial and marine fauna: a review. Aquat Bot.

[CR26] Nomann BE, Pennings SC (1998). Fiddler crab–vegetation interactions in hypersaline habitats. J Exp Mar Biol Ecol.

[CR27] Peterson G, Allen CR, Holling CS (1998). Ecological resilience, biodiversity, and scale. Ecosystems.

[CR28] Saintilan N, Williams RJ (1999). Mangrove transgression into saltmarsh environments in south‒east Australia. Global Ecol Biogeogr.

[CR29] Sheaves M, Brookes J, Coles R, Freckelton M, Groves P, Johnston R, Winberg P (2014). Repair and revitalisation of Australia’s tropical estuaries and coastal wetlands: opportunities and constraints for the reinstatement of lost function and productivity. Mar Policy.

[CR30] Shenker JM, Dean JM (1979). The utilization of an intertidal salt marsh creek by larval and juvenile fishes: abundance, diversity and temporal variation. Estuaries.

[CR31] Valiela I, Cole ML (2002). Comparative evidence that salt marshes and mangroves may protect seagrass meadows from land-derived nitrogen loads. Ecosystems.

[CR32] Valiela I, Bowen JL, York JK (2001). Mangrove Forests: One of the World’s Threatened Major Tropical Environments At least 35% of the area of mangrove forests has been lost in the past two decades, losses that exceed those for tropical rain forests and coral reefs, two other well-known threatened environments. Bioscience.

[CR33] Vermeiren P, Sheaves M (2014). Predictable habitat associations of four crab species across the low intertidal landscape of a tropical estuary over time. Estuar Coast.

